# A Network-Based Approach Exploiting Transcriptomics and Interactomics Data for Predicting Drug Repurposing Solutions Across Human Cancers

**DOI:** 10.3390/cancers17071144

**Published:** 2025-03-28

**Authors:** Alessio Galimi, Giulia Fiscon

**Affiliations:** 1 Department of Computer, Control, and Management Engineering “Antonio Ruberti”, Sapienza University of Rome, 00185 Rome, Italy; galimi.1665056@studenti.uniroma1.it; 2 Institute for Systems Analysis and Computer Science (IASI), National Research Council (CNR), 00185 Rome, Italy

**Keywords:** network theory, drug repurposing, pathways analysis

## Abstract

Background: Drug repurposing allows us to save time and money when looking for new therapeutic options. Providing a stronger foundation to support the drug proposals, it would result in faster and more reliable decision-making. Methods: We implemented a network-based algorithm, named PALADIN (Pathways Analyzer for off-LAbel inDIcatioNs), which combines a biological network of causal relationships, the modes of action, the target genes of the considered drugs, and transcriptomics data relative to various types of human cancers. Results: We identified several repurposable drug candidates, which we can understand at a deeper level thanks to the causal connections in the biological network. Conclusions: Many of the identified drug repurposing options are in accordance with the scientific literature about the drugs and their relationships with cancer, and by looking for the causal connections related to our top-rated drug–disease combination, we achieved a deeper understanding about the proposed candidates.

## Simple Summary

Drug repurposing can be an effective way to identify potential therapies for various diseases. Biological networks can be useful for this task, since they allow us to explore and analyze connections between biological entities in order to obtain new insights about them. In this work, we propose a network-based method to find drug repurposing solutions and visualize some of the biological mechanisms that support the repositioning proposals.

## 1. Introduction

According to the European Federation of Pharmaceutical Industries and Association (EFPIA), a drug takes about 12–13 years from the first synthesis of a new active substance for the medicinal product to reach the market [1]. The cost for the research and development of a new drug was estimated at USD 2558 million (in 2013 dollars) [2]. Moreover, on average, only one or two of every 10,000 substances will pass all the stages required to become a marketable medicine [1]. Where a new therapy is needed very quickly, the standard process to develop a new drug is simply too slow. If drug resistance arises for a particular disease, patients will die before a new treatment obtains approval [3]. In cases where there is not enough economic return for pharmaceutical companies, like for rare diseases, the companies will have trouble investing to develop medicines for those diseases. Moreover, each candidate drug might fail at any step of the process, regardless of how well the previous phases went.

A possible solution to all of these issues comes from the drug repurposing (DR) strategy. DR aims to find a new use for an already existing drug, outside of its original medical indication. The first successful examples of drug repurposing came through serendipity, but today, more scientific approaches are also possible [4]. To list some examples, aspirin, originally used as an analgesic, has been used clinically for at least 10 diseases, including coronary artery disease, Alzheimer’s disease, and colorectal cancer. New indications for aspirin are still being reported. Sildenafil, originally meant to treat cardiovascular disease, was later repositioned to cure erectile dysfunction [5]. Thalidomide’s story is almost dramatic: originally marketed as a sedative, it became very popular among pregnant women, thanks to its efficacy against morning sickness. When its teratogenicity effects showed up, its popularity fell to the ground. Several decades later, Thalidomide emerged again as a treatment for various inflammatory and malignant diseases, becoming in 2006 the first new treatment approved in over a decade for plasma cell myeloma [6].

There are huge cost and time savings when using drug repurposing and a much lower risk of failure. This is due to the fact that when repurposing a drug, a part of its research and development process has already been done. The preclinical testing is eliminated in repurposing projects [7], and for certain drugs, it may be possible to skip directly to Phase II of clinical trials [8].

Currently, various methods can be used to perform drug repurposing:

- Text-mining-based approaches. They involve extracting new knowledge from existing scientific literature [9].
- Semantic-based approaches. They integrate multiple sources (chemical, pharmacological, biological, biomedical, etc.) to build a semantic network, from which it is possible to predict new drug–disease relationships. While these methods have the advantage of making use of a massive number of databases, it is still a challenge to integrate all of these different sources [9].
- Transcriptional signature-based approaches. Transcriptomics data provide us with a list of over- and under-expressed genes in a biological system, treated with a drug or affected by a disease/condition. Comparing the transcriptional signatures can give us new insights on relationships between drugs and diseases [10].
- Molecular Docking. Here, we try to score the interaction between a small-molecule ligand and a protein to evaluate if a drug can bind to a new target [10].
- Network-based approaches. These methods organize the relationships between biological entities in the form of a network. Nodes can be many kinds of biological entities, like drugs, diseases, or proteins, while edges represent a direct or indirect interaction between two nodes [10]. A well-established algorithm falling in this category is SAveRUNNER [11], broadly applied in several biological contexts from viral infections to complex human diseases [12,13,14,15,16].

In our study, we performed drug repurposing by using a network-based approach, which extracts knowledge from multiple sources of omics data in order to predict putative repurposable candidates for treating different human cancer types. While most network-based methods have relied on the guilt-by-association principle, which hypothesizes that genes having similar function are located close together in the network, our approach is to use the network as a foundation to find causal chained connections that can provide an explanation about why a drug might be an effective repurposing candidate for a disease. This extra layer of significance can possibly speed up the process of validating the drug repurposing proposals.

## 2. Materials and Methods

### 2.1. Data Retrieval

#### 2.1.1. Gene Expression Data

The first type of input data is RNA-seq expression data, downloaded from the Xena platform [17] and preprocessed following the procedure adapted from [18], adapting it to our needs: we converted the genes’ identifiers from Ensembl ids to HUGO Gene Nomenclature (HGCN) ids, and we removed cancer types that did not have at least 20 normal samples and at least 20 tumor samples with expression not equal to zero. At the end of this preprocessing, we had samples from The Cancer Genome Atlas (TCGA) [19] and from the Genotype-Tissue Expression project [20] for 18 cancer types. In Table 1, the numbers of tumor samples and normal samples for each cancer type are summarized.

#### 2.1.2. Drug–Target Interactions

The other input data are the target proteins of the drugs. We retrieved these data from the Therapeutic Target Database (TTD) [21]. We also consider as input of the algorithm the mode of action (MoA) of the drug. There are various types of MoAs in the TTD, but we manually mapped them to only two types: “upregulation” and “downregulation”. We discarded MoAs with no direct mapping (for example, “modulation”).

#### 2.1.3. The SIGnaling Network Open Resource

SIGNOR [22] is a repository of manually annotated causal relationships between human proteins, chemicals of biological relevance, stimuli, and phenotypes. We retrieved a network of 10,066 nodes and 39,065 edges.

### 2.2. Preprocessing

#### 2.2.1. Differentially Expressed Genes Analysis

We first found the differentially expressed genes (DEGs) whose expression differs in a statistically significant way between the “normal” and “tumor” condition by exploiting the R package DESeq2 [23]. We adjusted for multiple-testing the resulting *p*-values by using the Benjamini–Hochberg method.

Differentially expressed genes (DEGs) are identified by applying a 0.05 threshold on the adjusted *p*-values, ending up with sets of DEGs for each cancer type.

#### 2.2.2. Disease Genes

We used the identified DEGs as disease-associated genes for each cancer type, filtering out all those genes not annotated in SIGNOR database. We then proceeded in creating cancer-specific networks of DEGs. By using the R package Hmisc [24], we computed Pearson’s correlations coefficients with relative *p*-values for each possible pair of genes, adjusting *p*-values using the Benjamini–Hochberg correction. We selected only edges with adjusted *p*-values lower than 0.05, which are also present in the SIGNOR-based graph. After this procedure, we obtained cancer-specific networks.

From these networks, we created a ranking of genes in order to select the most important ones to represent each disease. We leveraged the package SANTA [25], extending the procedure used in [18], by exploiting the packages iml [26] and caret [27] to refine the set of DEGs.

First, we assigned a weight to each node, given by multiplying the log2 fold-change (i.e., the logarithmic ratio between the averaged expression across tumor samples and the averaged expression across normal samples) by the node degree. Then, we normalized all the node weights to be in the range [0, 1], by using the formula:

(1) $$x_{norm}=\frac{x-weight_{min}}{weight_{max\;}-weight_{min}}$$

Furthermore, we computed a distance value for each couple of nodes, deriving it from the relative Pearson correlation coefficient *p*, with the following formula:

−*log10*(|*p*| + *c*) (2)

where *c* is a very small constant needed for numerical stability.

At this point, we used the knode function from the SANTA package to give a score to each node. We used this score to perform a first cut in our list of nodes. The knode score is given by the area under the curve of the following function:

(3) $$K_{i}^{node}=\frac{2}{n\bar{p}}\sum_{j}\left(p_{j}-\bar{p}\right)I\left(d^{g}\left(i,j\right)\leq s\right)$$

The weight of node *j* is given by $p_{j}$, while $\bar{p}$ is the average weight of all the nodes in the network. The number of nodes is given by *n*. We plotted this function for all values of distance, from 0 to the maximum distance value possible in the network. We should note that this function starts and ends at 0. Thanks to the identity function, at every distance value, only the nodes whose distance is equal or lower than the current distance value will contribute to the knode score. Nodes close to many “heavy” nodes will have the highest knode scores.

**Figure 1:**
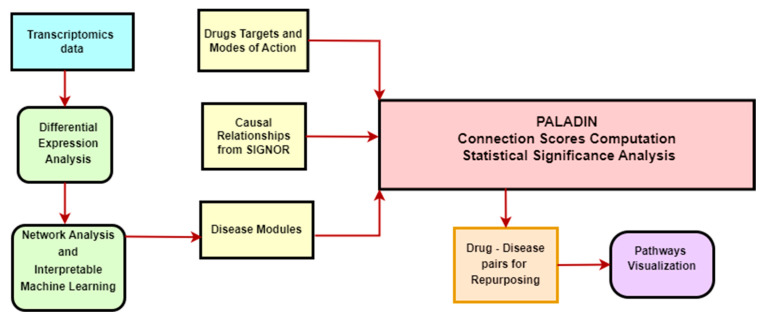
PALADIN’s workflow. Legend: in yellow, the input for PALADIN; in green, the preprocessing steps applied to the transcriptomics data; in red, the algorithm; in light blue, the transcriptomics data; in orange and violet, the Paladin’s output and the pathways visualization, respectively.

Depending on the number of samples we had for each cancer type, we selected the top 25, 50, or 100 genes by knode score. With the selected genes, we performed a classification task, using 5-fold cross validation on our samples. The aim of the classification problem is to use the information from the selected genes to distinguish normal tissue samples and tumor primary samples. We used a simple classification algorithm, K-Nearest Neighbors, to ensure that the performance of our model depends more on the features used than from a complex algorithm. We then applied the R package iml, whose acronym stands for Interpretable Machine Learning. We computed an important value for each feature by measuring the model prediction error before and after shuffling the values of the feature. When shuffling the values of the feature, we destroy the association between the feature and the prediction. The shuffling is repeated multiple times [28]. We selected only the genes with an importance value higher than zero. These genes were chosen as input for PALADIN (Figure 1).

By selecting the genes with this method, we observed an improvement to a simpler selection method like, for example, obtaining the top DEGs by absolute log2 fold-change. We compared the performance of a Radial Basis Function Support Vector Machine in performing the same binary classification task we did before, using as features the same numbers of genes, in the first case selected by importance values and in the second case selected by decreasing absolute log2 fold-change. The kappa statistic can be calculated using the following formula [29]:

(4) $$k=\frac{Pr\left(a\right)-Pr\left(e\right)}{1-Pr\left(e\right)}$$

*Pr*(*a*) represents the actual observed agreement between two raters (in our case, the prediction of our model and the ground truth). *Pr*(*e*) represents the expected chance agreement, which is the agreement we would expect to observe by chance. The chance agreement can be computed with the following formula starting from the confusion matrix [29]:

(5) $$\frac{\frac{{cm}^{1\;}\times \;{rm}^{1}}{n}+\frac{{cm}^{2}\;\times \;{rm}^{2}}{n}}{n}$$

The “*cm*” values represent the column 1 and column 2 marginals. The “*rm*” values represent the row 1 and row 2 marginals. The sample size is represented by *n*.

### 2.3. PALADIN—Functional Enrichment Analysis

The associations between disease genes and functional annotations such as KEGG pathways [30], Gene Ontology (GO) Biological Processes [31], and DisGeNET [32] were obtained by using EnrichR web tool [33]. *p*-values were adjusted with the Benjamini–Hochberg method (FDR), and a threshold equal to 0.05 was set to identify functional annotations significantly enriched amongst the selected gene lists. Graph visualization of the enrichment analysis as bipartite graph where nodes are genes and the functional categories of the different queried databases was obtained by using Enrichr-KG [34]. We present some of the visualizations in Section 3, while others, together with the tables containing the various analysis results, are available in the github repository.

### 2.4. PALADIN—Pathways Analyzer for Off-LAbel inDIcatioNs

**Figure 2:**
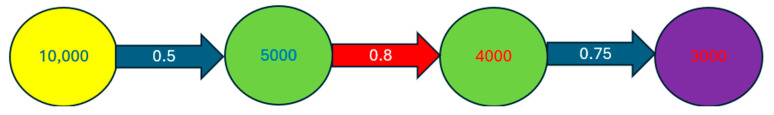
The source node is in yellow, and the target node is purple. The nodes that are part of the path are in green. The connections have a SIGNOR score associated with them. Connections are blue when they represent an upregulation and red when they represent a downregulation. The strength of the effect of the source node on the target node starts at an arbitrary value of 10,000. It gets reduced at each step by multiplying it with the SIGNOR score of the connection traveled. The strength of the effect also has a sign associated with it, which we choose to represent in this visualization using different colors: blue for positive sign and red for negative sign. The sign changes every time the path travels on a downregulation connection. In PALADIN’s R code, red values of strength are negative values.

The PALADIN algorithm works by navigating the SIGNOR network. SIGNOR is a directed network, with each edge having an associated SIGNOR score [35], representing the confidence in the validity of that relationship. Our first step is to calculate the *default connection scores*, between each node that can be a target for a drug and each node that we selected as representative for a disease. We found all the shortest paths going from a source node to a target node. For each of these paths, we multiplied a starting arbitrary value of 10,000 for the SIGNOR scores of the edges defining that path. We also considered the “effect” of each relationship (i.e., if the source node is suppressing or enhancing the target node), with the sign of the connection score changes, accordingly. At the end, a positive connection score means that if we upregulate the source node, we should have an upregulating effect on the target node, too. The absolute value of the connection score represents how strong this effect should be. We then summed the connection scores of each shortest path between two nodes to obtain the “default aggregate connection score” between the two nodes. We call it “default” because at this point, we are not considering the mode of action of the drugs on the source node. Starting with an arbitrary positive value is equivalent to imagining a default “activated” mode on the source node. We provide a visual representation of this computational process in Figure 2.

To compute the final connection score between a drug module and a disease module, we needed to consider each pair made by a drug target and a disease gene. We took the aggregate default connection score, reversed the sign if the mode of action of the drug was of type “suppression”, and reversed the sign again if the log2 fold change of the disease gene was negative. In this way, a negative sign of the connection score represents that if we use the drug on the considered drug target gene, the chain of effects should result in a final effect on the disease gene of the sign opposite to the alteration caused on the disease gene by the disease itself. For example, if a gene is overexpressed in a disease state, the drug should cause a chain of effects that leads to suppressing that gene. We then summed the connection scores of each pair to obtain the *final connection score* between a drug module and a disease module. We should note that if there is no connection between two nodes, that is equivalent to that path contributing to the final connection score with a score of zero.

We then proceeded to evaluate the statistical significance of the connection scores. Given the sizes of the drug modules and of the drug modules in our analysis, we randomly selected, for every drug size—disease size combination, 1000 randomly selected pairs of drug modules—disease modules of the same size as the original ones. The nodes that are candidates to be in a random drug module are all the drug targets, and the nodes that are candidates to be in a random disease module are all the disease genes. Then, we computed the connection scores for each built pair. With these 1000 connection scores, we computed a mean and a standard deviation to z-score normalize all the original connection scores originated from drug–disease pairs having the same sizes. We used the *p*-value to select the statistically significant connection scores (i.e., *p*-value ≤ 0.05). We should note that we are only looking for connection scores that are statistically significantly lower than expected.

## 3. Results and Discussion

### 3.1. PALADIN Analysis

**Figure 3:**
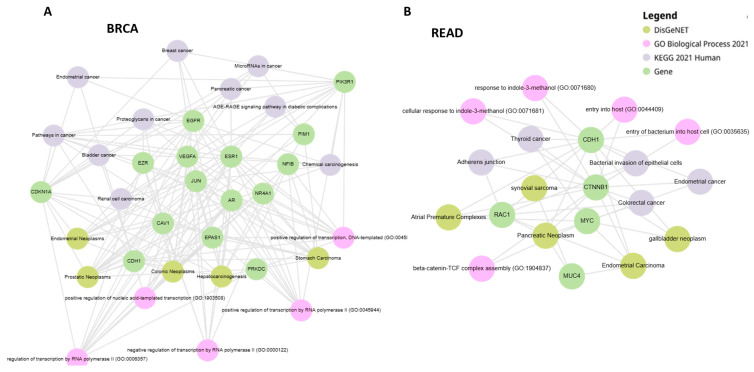
Knowledge graphs containing associations between GO Biological Processes, Kegg pathways, DisGeNET diseases, and the disease genes identified by PALADIN for, respectively, (**A**) BRCA, and (**B**) READ.

The PALADIN algorithm has been applied to study 18 cancer types. In particular, for each cancer type, we performed a comparison between the disease genes selected by the algorithm and a same-size group of DEGs selected by choosing the highest absolute log2 fold changes. For each cancer, the two groups of genes were used as features for a Radial Basis Function Support Vector Machine in order to perform a classification task aimed at distinguishing normal tissue samples and tumor primary samples. In Table 2, we report the performance of the two sets of genes, measured using AUC, accuracy, and kappa statistic. We highlight how the importance score seems to lead to a more significant group of genes as we highlight the results of functional enrichment analysis, showing important cancer-related pathways and disease–gene associations (Figure 3). BRCA genes are associated with various cancers and cancer-related pathways, including breast cancer, and the same is the case for READ genes, which are also associated with colorectal cancer.

In our study, we retrieved only drug–disease associations which are statistically significant according to our analysis, as can be seen in the *p*-value column in Table 3. We identified 66 statistically significant drug–disease relationships, all referring to Rectum Adenocarcinoma (Table 3). The disease module is the one relative to Rectum Adenocarcinoma. The 66 considered drugs cover a total of 13 different target genes.

In Table 3, there are 11 quinoline derivatives. Quinoline is an efficient scaffold for anticancer drug development as its derivatives have shown potent results through several mechanisms [36]. There are also 13 pyrazole derivatives, and it has been shown that many pyrazole derivatives have demonstrated multiple mechanisms of anticancer action by interacting with various targets [37].

We also find topiramate, which exhibits antitumorigenic and metastatic effects in ovarian cancer cells [38], and penfluridol, for which strong antitumor effects have been found in various cancer cell lines [39].

Trimethadione has been approved by the FDA for pancreatic cancer, and Mibefradil has been approved for the treatment of ovarian cancer, pancreatic cancer, and glioblastoma multiforme [40]. We should note that many of our proposed drugs are still in early stages of drug development, so they have a “Not Available (NA)” value in the FDA indication column.

**Figure 4:**
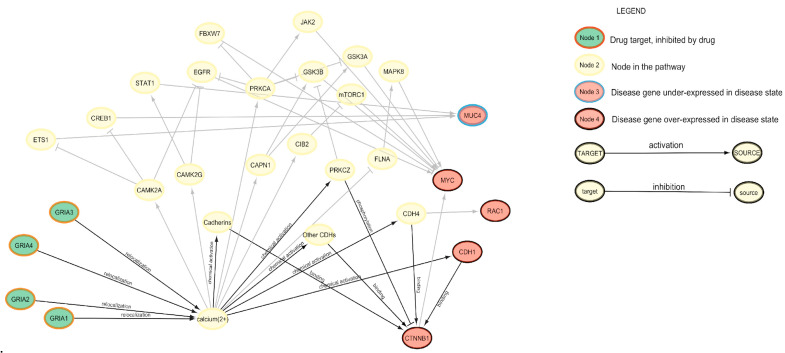
Pathways connecting the genes targeted by perampanel and the genes dysregulated the most by READ, with a focus on the most relevant pathways according to PALADIN. The network visualization was performed via Cytoscape version 3.10.3 [42].

The strongest drug repurposing proposal according to the connection scores is perampanel, an anticonvulsant which exhibited antitumoral effects, such as cell viability inhibition and apoptosis induction [41]. In Figure 4, the connections between the genes targeted by perampanel and the genes representative for Rectum Adenocarcinoma are visualized.

We observed that the drug has a suppressing effect on various types of subunits of Glutamate Ionotropic Receptor AMPA, the predominant neurotransmitter receptors at central synapses [43]. Ca^2+^ is arguably the most important second messenger in the brain because of its pivotal roles in presynaptic neurotransmitter release, postsynaptic responses, and plasticity induction. Ionotropic GluRs and metabotropic GluRs can generate intracellular Ca^2+^ signals. The proteins involved in regulating Ca^2+^ signaling are often remodeled in tumor cells to sustain proliferation and avoid cell death [44]. It seems reasonable to see these proteins as a potential therapeutic strategy to be explored.

### 3.2. SAveRUNNER Analysis and Comparison

We compared the results obtained by our algorithm with the ones we obtained using another well-established tool for network-based drug repurposing, SAveRUNNER [11]. The three input types needed by the two algorithms are the same: human interactome, drug targets, and disease genes. We fed SAveRUNNER with the same disease-specific genes that we selected for PALADIN. The drug target datasets used come from DrugBank [45] for SAveRUNNER and from the Therapeutic Target Database for PALADIN, different due to the fact that PALADIN requires the mode of action information to function. Regarding the interactomics data, SAveRUNNER uses an undirected network [46] that combines 21 public PPI databases, all compiling experimentally derived protein–protein interactions; while PALADIN uses SIGNOR, a smaller but directed network. SAveRUNNER implements a novel network similarity measure that rewards drug and disease genes falling in the same network neighborhood, detailed in [11,12]. Specifically, for each drug target genes, SAveRUNNER computes the shortest path to the disease genes set, and then it takes the average shortest path for all the drug target genes. Note that SAveRUNNER requires at least 95% of the drug targets to be able to reach the disease genes through the network; otherwise, the two modules are considered disconnected. To evaluate the statistical significance of these proximity values, the software builds a reference distance distribution by randomly selecting two groups of proteins in the interactome, with the same sizes and degree distributions of the original sets, and computes the proximity between them. This procedure is repeated 1000 times to build the distance distribution. SAveRUNNER uses the mean and the standard deviation from this distribution to z-score normalize the original observed proximity value, obtaining a *p*-value. Using this *p*-value, it is possible to select only the statistically significant drug–disease interactions (*p*-value ≤ 0.05).

By running SAveRUNNER for all the analyzed datasets, we obtain 2600 drug–disease combinations (Supplementary Table S1). By comparing the results with PALADIN’s output, we found hesperidin as a drug predicted as a potential candidate for the treatment of READ. Of note, hesperidin showed in vitro efficacy in suppressing colorectal cancer [47]. We also found four other drugs between those predicted by PALADIN and those being proposed by SAveRUNNER for the treatment of other cancers: ethosuximide for KIRP and UCEC; verapamil for LUSC, STAD, and UCEC; trimethadione for KIRP and UCEC; and methsuximide for KIRP, LUAD, and UCEC.

**Figure 5:**
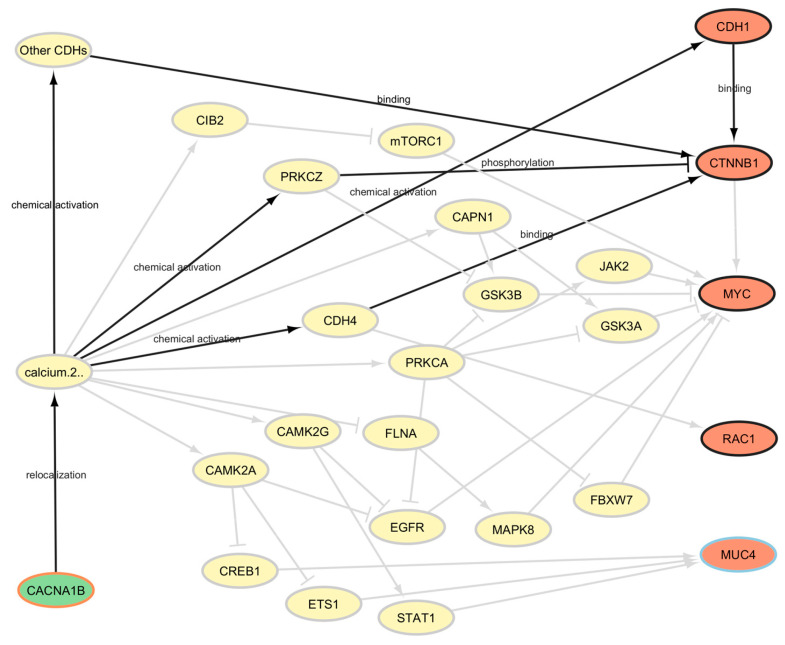
Pathways connecting the genes targeted by hesperidin and the genes dysregulated the most by READ, with a focus on the most relevant pathways according to PALADIN.

Comparing the results of the two algorithms allows us to restrict the field of repurposing candidates. PALADIN also provides us with an explanation of the repurposing proposal: in Figure 5, we show the causal pathways connecting hesperidin with READ as they were identified by PALADIN.

Evidence in the literature showed CACNA1B being overexpressed in prostate and breast cancer tissues when compared to adjacent normal tissues, and this gene’s overexpression appears to be an independent prognostic marker for non-small-cell lung cancer in the Chinese population. The function of CACNA1B (Cav2.2) is tightly linked to tumor intracellular Ca^2+^ concentration, and targeting intracellular calcium levels through the N-type voltage-gated calcium channel encoded by CACNA1B might represent a novel therapy for NSCLC [48].

## 4. Conclusions

In this study, we used transcriptomics data to identify genes dysregulated in disease states, drug information to select the genes targeted by the drugs and to categorize their mode of action, and a biological network based on directed, causal relationships to identify the shortest paths connecting drugs and diseases. The SIGNOR scores helped us obtain a quantitative estimate of the effect of each drug of each disease, and thanks to the statistical significance of the results, we were able to deeply investigate the pathways going from drug targets to a disease module to better understand the mechanisms through which the drug might exercise its therapeutic effect. Having a drug repurposing approach with a causality-based focus might help researchers to be a step ahead when evaluating drug repositioning proposals and trying to validate them.

We identified some drugs as repurposing candidates for Rectum Adenocarcinoma, whose potential is supported by the scientific literature. We looked at the causal relationships contained in SIGNOR between perampanel’s (i.e., the top ranked repurposing proposal) target genes and genes significantly affected by READ, and we did the same for hesperidin, a drug that was predicted by both PALADIN and SAveRUNNER as repurposable for READ. In both cases, we appreciated the explainability of the drug mechanisms that this new approach made possible.

Of course, this study is limited by its computational nature, offering testable hypotheses that have to be tested and may eventually be translated into clinical applications.

As a future perspective, more biological insights might be added to the analysis, for example, by considering the possible side effects of a drug.

## Figures and Tables

**Table 1:** Tumor and normal samples for each cancer type.

**Normal Samples**	**Tumor Samples**	**Cancer Acronym**	**Cancer Type**
101	180	UCEC	Uterine corpus
			Endometrial carcinoma
339	119	THYM	Thymoma
338	512	THCA	Thyroid carcinoma
211	413	STAD	Stomach adenocarcinoma
557	468	SKCM	Skin cutaneous melanoma
317	92	READ	Rectum adenocarcinoma
151	495	PRAD	Prostate adenocarcinoma
171	179	PAAD	Pancreatic adenocarcinoma
338	498	LUSC	Lung squamous
			Cell carcinoma
347	513	LUAD	Lung adenocarcinoma
160	369	LIHC	Liver hepatocellular
			Carcinoma
60	288	KIRP	Kidney renal papillary cell carcinoma
100	530	KIRC	Kidney renal clear cell carcinoma
53	66	KICH	Kidney chromophobe
286	182	ESCA	Esophageal carcinoma
348	288	COAD	Colon adenocarcinoma
291	1099	BRCA	Breast cancer
28	407	BLCA	Bladder cancer
4196	6698		TOTAL

**Table 2:** Performance comparison of a Support Vector Classifier with Radial Basis Function by AUC, accuracy, and Cohen’s kappa, using as features, in the first case, the top DEGs by log2fold-change, and in the second case, the significant DEGs selected by the procedure involving knode score and importance score. Each couple of columns shows the performance of a metric for the two gene sets. The higher value for each metric and for each cancer is in bold.

Disease	Kappa log2fc	Kappa Sign Genes	Accuracy log2fc	Accuracy Sign Genes	AUC log2fc	AUC Sign Genes
KICH	0.8799	**0.966**	0.9403	**0.9832**	0.9841	**0.9994**
BLCA	0.1423	**0.3485**	0.9352	**0.9386**	**0.9471**	0.8906
BRCA	0.9428	**0.9539**	0.9809	**0.9847**	0.9974	**0.9987**
COAD	0.9169	**0.974**	0.9588	**0.9871**	0.992	**0.999**
ESCA	**0.9277**	0.9005	**0.9654**	0.9521	0.983	**0.9913**
KIRC	0.7563	**0.9253**	0.9251	**0.9803**	0.9783	**0.9923**
KIRP	0.7814	**0.9208**	0.9282	**0.9776**	0.9854	**0.997**
LIHC	**0.8763**	0.8351	**0.9474**	0.931	**0.989**	0.9756
LUAD	0.931	**0.9623**	0.967	**0.9819**	0.9969	**0.9979**
LUSC	0.9501	**0.9675**	0.9761	**0.9843**	**0.997**	0.996
PAAD	0.8634	**0.9514**	0.9317	**0.9757**	0.9861	**0.989**
PRAD	0.6914	**0.8429**	0.8872	**0.9446**	0.9046	**0.9781**
READ	0.7492	**0.8917**	0.8985	**0.9601**	0.9888	**0.9971**
SKCM	0.9418	**0.9736**	0.971	**0.9869**	0.9947	**0.997**
STAD	**0.9255**	**0.9255**	0.9665	**0.967**	**0.9943**	0.9939
THCA	**0.9315**	0.8763	**0.9673**	0.9408	**0.9937**	0.9901
THYM	**0.9887**	0.9543	**0.9956**	0.9825	**0.999**	0.9976
UCEC	**0.9572**	0.9259	**0.9804**	0.9655	0.9981	**0.9984**

**Table 3:** The most significant drug–disease relationships computed by PALADIN.

Drug TTD ID	Distance to READ Module	FDA Indication	*p*-Value	Drug Names
D0U3ED	-5,660,390	Diabetic neuropathy	0.030376	E-2007
D01EVT	-1,436,887	NA	0.03374	Ethinyl-pyrazole derivative 1
D02RRE	-1,436,887	NA	0.03374	Pyrazole derivative 78
D03KYL	-1,436,887	NA	0.03374	Quinoline derivative 4
D03PRA	-1,436,887	NA	0.03374	Heteroaryl-pyrazole derivative 2
D04LRT	-1,436,887	NA	0.03374	Quinoline derivative 9
D04VAE	-1,436,887	NA	0.03374	PMID25435285-Compound-26
D05CBS	-1,436,887	NA	0.03374	PMID25435285-Compound-37
D06NZY	-1,436,887	NA	0.03374	PMID25435285-Compound-44
D08JPW	-1,436,887	NA	0.03374	N-substituted pyrazole derivative 1
D08PHH	-1,436,887	NA	0.03374	PMID25435285-Compound-45
D09JZA	-1,436,887	NA	0.03374	Quinoline derivative 8
D09NRH	-1,436,887	NA	0.03374	Quinoline derivative 5
D09OQZ	-1,436,887	NA	0.03374	PMID25435285-Compound-49
D09WXH	-1,436,887	NA	0.03374	PMID25435285-Compound-25
D0B6DN	-1,436,887	NA	0.03374	Ethinyl-pyrazole derivative 3
D0BP6R	-1,436,887	NA	0.03374	Ethinyl-pyrazole derivative 2
D0C2ZJ	-1,436,887	NA	0.03374	Tetra-hydro-imidazo[1,5-d][1,4]oxazepin-3-yl derivative 3
D0CZ6T	-1,436,887	NA	0.03374	Tetra-hydro-imidazo[1,5-d][1,4]oxazepin-3-yl derivative 2
D0E5QO	-1,436,887	NA	0.03374	Quinoline derivative 7
D0H2BU	-1,436,887	NA	0.03374	PMID25435285-Compound-53
D0H2LD	-1,436,887	NA	0.03374	PMID25435285-Compound-43
D0HD8F	-1,436,887	NA	0.03374	Pyrazole derivative 76
D0HG1W	-1,436,887	NA	0.03374	2-(substituted ethynyl)quinoline derivative 4
D0I6LO	-1,436,887	NA	0.03374	PMID25435285-Compound-47
D0IL4Z	-1,436,887	NA	0.03374	PMID25435285-Compound-38
D0IY8T	-1,436,887	NA	0.03374	PMID25435285-Compound-46
D0K0ZX	-1,436,887	NA	0.03374	Heteroaryl-pyrazole derivative 3
D0K1RU	-1,436,887	NA	0.03374	Quinoline derivative 3
D0KZ6Q	-1,436,887	NA	0.03374	PMID25435285-Compound-40
D0LI2E	-1,436,887	NA	0.03374	Tetra-hydro-imidazo[1,5-d][1,4]oxazepin-3-yl derivative 1
D0MB0V	-1,436,887	NA	0.03374	2-(substituted ethynyl)quinoline derivative 2
D0N0FA	-1,436,887	NA	0.03374	PMID25435285-Compound-51
D0N1CC	-1,436,887	NA	0.03374	PMID25435285-Compound-42
D0N9IA	-1,436,887	NA	0.03374	Tetra-hydro-imidazo[1,5-d][1,4]oxazepin-3-yl derivative 4
D0NE0W	-1,436,887	NA	0.03374	Tetra-hydro-imidazo[1,5-d][1,4]oxazepin-3-yl derivative 6
D0O6QC	-1,436,887	NA	0.03374	Heteroaryl-pyrazole derivative 1
D0O9TL	-1,436,887	NA	0.03374	PMID25435285-Compound-52
D0OT4O	-1,436,887	NA	0.03374	Quinoline derivative 6
D0P7DA	-1,436,887	NA	0.03374	PMID25435285-Compound-10
D0PJ8X	-1,436,887	NA	0.03374	2-(substituted ethynyl)quinoline derivative 1
D0Q2FC	-1,436,887	NA	0.03374	Tetra-hydro-imidazo[1,5-d][1,4]oxazepin-3-yl derivative 5
D0QM1R	-1,436,887	NA	0.03374	Pyrazole derivative 77
D0RX0M	-1,436,887	NA	0.03374	PMID25435285-Compound-39
D0S5SK	-1,436,887	NA	0.03374	Pyrazole derivative 79
D0T3DG	-1,436,887	NA	0.03374	BCI-632
D0V0BC	-1,436,887	NA	0.03374	N-substituted pyrazole derivative 2
D0WP5S	-1,436,887	NA	0.03374	N-substituted pyrazole derivative 3
D0XQ4T	-1,436,887	NA	0.03374	2-(substituted ethynyl)quinoline derivative 3
D0Y5DN	-1,436,887	NA	0.03374	PMID25435285-Compound-41
D0YZ7D	-1,436,887	NA	0.03374	PMID25435285-Compound-50
D00GEG	-1,415,097	NA	0.035841	Ralfinamide
D01NLB	-1,415,097	Pain	0.035841	Ziconotide
D07VDZ	-1,415,097	Epilepsy; alcohol use disorders	0.035841	Topiramate
D08EOD	-1,415,097	Epileptic seizures	0.035841	Methsuximide
D09JBP	-1,415,097	Paramethadione syndrome; seizures	0.035841	Paramethadione
D0A3MJ	-1,415,097	NA	0.035841	XEN007
D0CQ0Z	-1,415,097	Schizophrenia	0.035841	Penfluridol
D0I9HF	-1,415,097	Capillary fragility	0.035841	Hesperidin
D0M8AB	-1,415,097	Dietary shortage	0.035841	Glycine
D0N3SR	-1,415,097	NA	0.035841	Cilnidipine
D0Q4XQ	-1,415,097	Epilepsy	0.035841	Ethosuximide
D0R0FE	-1,415,097	Hypertension; angina	0.035841	Verapamil
D0U4VT	-1,415,097	Epileptic conditions; pancreatic cancer	0.035841	Trimethadione
D0U7GP	-1,415,097	NA	0.035841	Rauwolfia Serpentina root
D0W8XT	-1,415,097	Granted orphan drug status by FDA for ovarian cancer, pancreatic cancer, and glioblastoma multiforme	0.035841	Mibefradil

## Data Availability

The R code to reproduce this work is available at https://github.com/Alessio-Galimi/PALADIN (accessed on 19 March 2025). Most of the datasets obtained during the execution of PALADIN’s workflow have been also uploaded to https://github.com/Alessio-Galimi/PALADIN. The rest of the data can be obtained by executing the R code. The input data is available online as described in Section 2.

## Supplementary Materials

The following supporting information can be downloaded at: https://www.mdpi.com/article/10.3390/cancers17071144/s1, Table S1: drug–disease network provided by SAveRUNNER analysis, providing the repurposable candidates for all the analyzed diseases.

## Abbreviations

The following abbreviations are used in this manuscript:

PALADIN	Pathways Analyzer for off-LAbel inDIcatioNs
DR	Drug Repurposing
TCGA	The Cancer Genome Atlas
HGCN	HUGO Gene Nomenclature
UCEC	Uterine Corpus Endometrial Carcinoma
THYM	Thymoma
THCA	Thyroid carcinoma
STAD	Stomach Adenocarcinoma
SKCM	Skin Cutaneous Melanoma
READ	Rectum Adenocarcinoma
PRAD	Prostate Adenocarcinoma
PAAD	Pancreatic Adenocarcinoma
LUSC	Lung squamous cell carcinoma
LUAD	Lung Adenocarcinoma
LIHC	Liver Hepatocellular Carcinoma
KIRP	Kidney renal papillary cell carcinoma
KIRC	Kidney renal clear cell carcinoma
KICH	Kidney chromophobe
ESCA	Esophageal carcinoma
COAD	Colon adenocarcinoma
BRCA	Breast cancer
BLCA	Bladder cancer
SAveRUNNER	Searching off-lAbel dRUg aNd NEtwoRk
SIGNOR	SIGnaling Network Open Resource
DEGs	Differentially Expressed Genes
SANTA	Spatial Analysis of Network Associations
iml	Interpretable Machine Learning
